# Numerical Analysis of Bowing Phenomenon Due to Thermal Stresses in Marble Slabs

**DOI:** 10.3390/ma13194367

**Published:** 2020-09-30

**Authors:** William Hideki Ito, Anna Maria Ferrero, Paulo Ivo Braga de Queiroz

**Affiliations:** 1 Department of Earth Sciences, University of Turin, 35 Valperga Caluso Street, 10125 Turin, Italy; anna.ferrero@unito.it; 2 Division of Civil Engineering, Aeronautics Institute of Technology; 50 Marechal Eduardo Gomes Square, 12228-900 São José dos Campos, Brazil; pi@ita.br

**Keywords:** geometric nonlinearity, FEM, thermoelasticity, bowing, transient heat flux

## Abstract

Bowing is a pathology known by the deformation experienced in some external covering systems in ornamental stones, especially in marble, and thermal action is one of the key factors that lead to this degradation. Previous studies presented remarkable contributions about the mechanical behavior of bowing but they were based on classical beam’s theory and improper assumptions might mislead the evaluation of internal stresses. This study proposes to evaluate internal stresses in bowing due to thermal loading considering the true deformed shape in continuum media. Finite displacement concepts are proposed to calculate stress-strain relationship and comparison with linear elastic theory is also addressed. Internal stresses not predictable in the Euler-Bernoulli beam were found in parametric analyses. Moreover, the numerical analysis accomplished in this paper indicates that transient heat flux should induce higher stresses than just considering higher gradients of temperature in steady flux which could explain the larger decohesion through width in bowing tests.

## 1. Introduction

Nonlinear mechanics theory has once been strictly applied in special problems in engineering, such as aeronautical and mechanical fields, due to higher computational effort and accuracy required in those analyses. Other areas have used small strain approach because it could well represent the great majority of problems adopting simplified hypotheses. However, the advances in technology have allowed widespread uses of nonlinear models in some areas where small strain was predominant, such as geotechnical and rock engineering, where assumption of small deformations, in deep underground excavation or landslides, might not be respected anymore [1,2,3,4].

In accordance with this trend it is proposed in this work to evaluate the effect of geometric nonlinearity and internal stress development in a specific problem in rock mechanics known as bowing. It can be defined as a pathology that affects some kind of marbles and causes permanent deformation due to weathering of these ornamental stones when used in external environment. In ventilated façade the deformation of marble slabs might reach large displacement easily noticed visually (see [5,6]) and similar decay might be responsible to deteriorate some sculptures in marble.

Thermal load due to sunlight exposure was found as an important factor to contribute to this degradation and previous studies of bowing in marble were mainly focused to understand the micromechanical phenomenon trying to simulate the intergranular decohesion due to anisotropic thermal expansion of calcite grains. Royer-Carfagni [7] presented an initial discussion about induced shear stresses by thermal actions in a chessboard like mineralogical arrangement. After that, Weiss et al. [8] improved that analysis using finite element analysis (FEA) to evaluate the stress distribution in microscale using the contours of calcite grains from a thin section image. One of the first attempts to represent the macrophenomenon was proposed by Ferrero et al. [9], improved later by Ferrero et al. [10]. These works presented models to predict the macromechanical behavior through linear elastic fracture mechanics considering the Euller–Bernoulli beam theory.

Beam’s theories, such those used in previous studies, presume deformed shape which will fit to equilibrium equations. For instance, in Euler-Bernoulli beam the orthogonality of planes in relation to neutral axis in reference and deformed configuration are assumed, i.e., according to this theory the material is infinitely rigid to distortion by shear stresses. Linear and third order approaches considering shear strain were proposed by Timoshenko and Reddy [11] to consider this effect in thick beams; nonetheless, deformation is still imposed by these hypotheses.

Since the thermal gradient in a ventilated façade is relatively small to generate great internal stresses if were compared to those in thermal machines (turbines, diesel engines, etc.) it is proposed to evaluate the problem considering a more refined solution. A different approach could be solving the differential equations of equilibrium respecting the constitutive relationship and compatibility, which means that the equilibrium in deformed shape will occur according to the displacement field that minimizes the total potential energy, just as predicted in variational theorems. Advantages over previous studies are based on the true deformed shape since no restriction to deformation is previously imposed.

Inexact solution considering linear elasticity could also be reviewed to get more accurate solution; hence it is proposed to evaluate the problem via FEA considering geometric nonlinearity. Different ways could be chosen to approach the problem, such as, Cosserat continuum, true (logarithmic) strain or corotational formulation [12,13]. Herein will be considered the updated Lagrangian formulation using polar decomposition to overcome the approximations assumed in linear elastic theory. Nonlinear concepts are applied to evaluate transient thermal loads in marble slabs and a MATLAB code was implemented to compute the development of internal stresses via finite element method (FEM), including pre and post processing analyses. The formulation herein proposed presents relevant contribution not only to the understanding of bowing in marble slabs but also in other fields, for instance, the influence of nonlinear thermoelasticity in thin membranes [14,15] since the consideration of thermal stresses at micro and nanoscale, respectively, is important to understand the behavior of these materials.

## 2. Review of Bowing

Despite the fact that degradation of ornamental stones has long been studied in cultural heritage field mainly focused on preservation of monuments, historical heritages and statues [16,17,18,19,20], bowing was identified just few decades ago and has degraded some specific kinds of ornamental stones in short periods. Several types of marbles and limestones from different countries were affected by this degradation process, although it affects the most calcitic marble. White Carrara marble is an example of ornamental stone affected and the one more frequent in literature about this subject. It comes from the Alpi-Apuane complex formed by metamorphic processes in limestone deposits and can be considered a monomineralic rock composed mainly by calcite.

Many efforts were made to understand this complex phenomenon but still remains some open questions about it. The major research projects about this theme were MARA [21] and TEAM [22] which produced good insight into this problem. Some hypotheses proposed by Winkler [23] were verified in laboratory tests, such as the influence of thermal anisotropy and the microstructure, while other aspects are not yet quite understood.

One of the most known cases was verified at the Finland Hall’s façade in which bowing affected the external covering system twice. According to Gelk et al. [24], the first time the panels were composed by white Carrara marble fixed at vertical joints by dowels and the largest ones were 140 cm length and 3 cm thickness. Just some years later the deflection of bowed panels reached up to 5 cm inward relative to the façade (concave). In the renewal façade, though, bowing occurred outward (convex). Similar problems were also verified in other buildings spread in Europe and North America [22]. Siegesmund et al. [6] verified correlation between height of the buildings and deflection of bowed slabs, even if it were not possible to apply for general cases.

Thermal anisotropy of calcite grains has been considered as one of the major causes responsible to contribute to this degradation since that mineral is the primary component of limestones and marbles. Calcite belongs to the hexagonal crystal family and the thermal expansion coefficients aligned with these optical axes present antagonist behavior. When a calcite grain is submitted to heating load it tends to expand in C-axis direction, but contracts in A-axis direction, which generates internal stresses during heating-cooling cycles.

Gelk et al. [25] verified that cyclic thermal loading could cause some deformation on marble specimens, nonetheless, it is extremely influenced by humidity. Plastic deformation rate is larger at the first cycles and decreases. In dry environments the residual deformation stabilizes after some heating-cooling cycles. On the other hand, in those marble specimens tested in wet environment, the residual deformation could increase continuously. Similar results were found by Koch and Siegesmund [26], Rodriguez-Gordillo and Saez-Perez [27], Luque et al. [28] and others. Bellopede et al. [29] verified that artificial weathering could be used to represent the natural one in accelerated way. These authors also verified that the decohesion of calcite grains increases from the less to the more weathered faces, which agrees with deformed shapes of weathered marbles.

Correlation between microstructure and bowing were also found in previous studies. Regular structures (granoblastic texture) tend to bow more than irregular (xenoblastic) ones. Influence of locked-in stresses due to metamorphic process, exploitation procedures, external forces applied by supporting anchorage systems can also be cited as contribution factors.

## 3. Fundamentals

Typical problems in rock engineering are usually dealt with engineering strain because rocks are assumed as brittle materials and small deformations are expected previous failure. Engineering strain, or small strain, assumes some linearities to approach stress-strain relationship, however, cases in which deformation becomes non infinitesimal are not so uncommon. Limitation previously imposed by small strain might lead to inexact evaluation when the hypothesis of infinitesimal deformation no longer occurs, or refined approach is required.

Even considering the small gradient of temperature between internal and external faces in ventilated façades studies have shown the importance of these induced stresses in bowing. For this reason, it is proposed to study the problem using nonlinear concepts, which take into account the continuous deformation of a body and due to corrections at each step even large deformations can be assessed.

### 3.1. Finite Displacement Concepts

In continuum mechanics the deformation gradient (***F***) is used to express the current configuration in relation to a reference state. There are several ways to represent this tensor and a useful one to understand it could be in terms of principal stretches and principal directions as presented in Equation (1), in which λ represents the stretch in each principal direction. Due to polar decomposition it can be decomposed as the product of a pure rotational (***R***) and a pure stretch tensor (U or ***V***). Right polar decomposition considers that stretch of fibers occurs first and then rotation tensor is applied; in the left polar decomposition the order of deformation is changed.

(1) $$F=\overset{3}{\underset{i=1}{\sum }}\lambda_{i}e_{i,x}⊗e_{i,X}$$

Green–Lagrange strain is defined as the quadratic changing of a fiber prior to its rotation and can be expressed in terms of deformation gradient as given by Equation (2). Since the stretches are rotated, it can be used in problems of finite deformation without the approximation assumed in small strain theory [30]. Substituting ***F*** by the product of right polar decomposition in Equation (2) one can get to Equation (3) which proves that rotation does not affect this strain measure.

(2) $$2\;\epsilon =F^{T}F-I$$

(3) $$2\;\epsilon ={\left(RU\right)}^{T}RU-I=U^{T}\left(R^{T}R\right)U-I=U^{2}-I$$

The energetic conjugate of Green strain is the 2nd Piola–Kirchhoff stress tensor. It does not have direct physical interpretation such as Cauchy stresses because it considers stresses acting on the reference configuration rotated to the current state, but it can be related with real stress as given in Equation (4) [30]:

(4) $$S=\left|F\right|\;F^{-1}\;\sigma \;F^{-T}$$

Since these stress-strain measures are energetic conjugate pairs, the product of these tensors produces a scalar quantity, energy per volume, such as the integration using Cauchy stress and real strain tensors, as indicated by Equation (5). It must be pointed out that the left-hand side is integrated over the reference configuration, i.e., known shape, and the other is related with the deformed shape.

(5) $$\int_{\Omega_{0}}S\;\delta \epsilon d\Omega_{0}=\int_{\Omega }\sigma \;\delta \varepsilon d\Omega$$

### 3.2. Constitutive Model

As described in the previous section, the correlation between deformation due to weathering and the influence of some factors which induce bowing are known, but the real mechanisms are not yet fully understood. Internal stresses developed by thermal gradient have shown as an important factor acting on bowing, thus, it will be the object of this study. Since the purpose of this work remains on the evaluation of induced strains it is proposed a 2-D solid formulation in plane strain state with homogeneous characteristics.

According to theory of elasticity no stress should be induced in a continuous homogeneous isotropic material subject to uniform temperature increasing. Nonetheless, gradient of temperature occurring in the inner part of a body would induce stress due to differential strain [31,32,33]. In such analysis the total strain could be decomposed in thermal and mechanical strain, in which the last one would be responsible for induce internal stresses. Then, the total strain is given by:

(6) $$\epsilon =\epsilon_{\theta }+\epsilon_{mec}$$

Assuming St. Venant-Kirchhoff nonlinear material, internal stresses in bowing could be calculated via the constitutive stress-strain relationship written in Equation (7):

(7) $$S=C\;\epsilon_{mec}=C\;\left(\epsilon -\epsilon_{\theta }\right)$$

where

$$C=A\left(\begin{array}{ccc} 1-\nu^{\prime } & \nu & 0\\ \nu^{\prime } & 1-\nu^{\prime } & 0\\ 0 & 0 & \frac{1}{2}-\nu^{\prime } \end{array}\right),\;A=\frac{E^{\prime }}{\left(1-2\nu \right)\left(1+\nu \right)},E^{\prime }=\frac{E}{1-\nu^{2}}\;\;\mathrm{and}\;\nu^{\prime }=\frac{\nu }{1-\nu }$$

**Figure 1:**
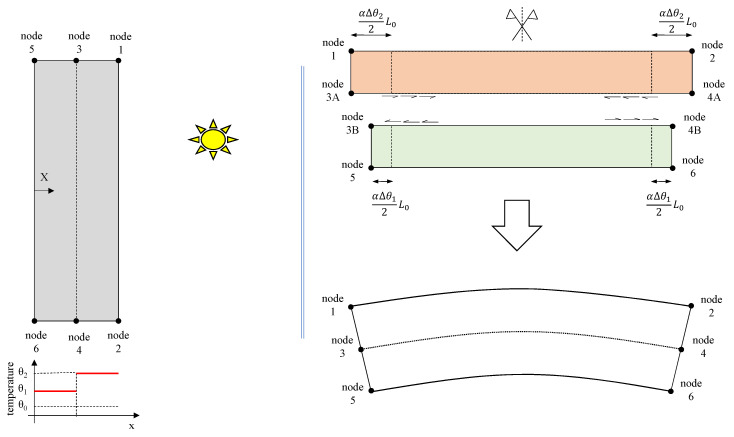
Idealization of the deformation due to thermal loading in continuum media.

For sake of simplicity, one considers a coarse discretization of a slab laid on the ground subject to sunlight exposure on the external face, as illustrated in Figure 1. In this example the deformation will be limited only to y direction and uniform increase of temperature inside each element is assumed. According to classical elastic theory, for a generic material with positive thermal expansion coefficient, the right-hand side should present larger expansion than the other. However, due to compatibility, the displacements of pairs 3A–3B and 4A–4B must coincide, therefore, shear stresses on this interface should induce compressive stress at the upper element and tensile stresses at the lower. It is clear that the shear stress along the interface must not be constant once it should be zero at the boundary (nodes 3 and 4) and at the symmetry plane. The same concept can be applied to a more refined mesh and to other directions.

In continuum media the stress-strain relationship could be solved for a displacement field which satisfies the equilibrium, compatibility and constitutive relationship by the principle of virtual work. Considering that only mechanical strain should induce stresses in the body, the variation of total potential energy can be described as presented in Equation (8), and the equivalent internal and external works as follow in Equations (9) and (10), respectively.

(8) $$\delta \Pi =\int_{\Omega_{0}}\;\delta \epsilon_{v}{}^{T}C\;\left(\epsilon -\epsilon_{\theta }\right)d\Omega_{0}-\int_{\Omega_{0}}\delta u_{v}^{T}\;f^{B}d\Omega_{0}-\int_{\Gamma_{0}}\delta u_{v}^{T}\;f^{S}d\Gamma_{0}$$

(9) $$\delta W_{int}=\int_{\Omega_{0}}^{}\;\delta \epsilon_{v}{}^{T}C\;\epsilon d\Omega_{0}$$

(10) $$\delta W_{ext}=\int_{\Omega_{0}}^{}\;\delta E_{v}{}^{T}C\;\left(\epsilon_{residual}\right)d\Omega_{0}+\int_{\Omega_{0}}\delta u_{v}^{T}\;f^{B}d\Omega_{0}+\int_{\Gamma_{0}}\delta u_{v}^{T}\;f^{S}d\Gamma_{0}$$

## 4. Numerical Implementation

Using the discretization via FEM, the displacement field in a continuum media is described by interpolation of nodal displacement according to specifics shape functions:

(11) $$u=\sum^{\;}N_{i}\left(\xi ,\eta \right)\;\tilde{u}$$

Deformation gradient and the Green strain tensor are given by [34]:

(12) $$F_{IJ}=\delta_{iI}+\frac{\partial u_{i}}{\partial X_{I}}$$

(13) $$\epsilon_{IJ}=0.5\left(\delta_{iI}\frac{\partial u_{i}}{\partial X_{J}}+\delta_{iJ}\frac{\partial u_{i}}{\partial X_{I}}+\frac{\partial u_{i}}{\partial X_{I}}\frac{\partial u_{i}}{\partial X_{J}}\right)$$

where *δ_iI_* and *δ_iJ_* are Kronecker delta

Finite strain equations are solved similarly as in small strain problems but considering the reference configuration instead. Through Newton–Raphson procedure, the variation of internal work is calculated by increments of displacements that minimize the variation of total energy, then, the new value is updated from previous step:

(14) $$\delta \delta W_{int}^{t+\Delta t}=\delta W_{ext}^{t}-\delta W_{int}^{t}$$

(15) $$\delta W_{int}^{t+\Delta t}=\delta W_{int}^{t}+\delta \delta W_{int}^{t+\Delta t}$$

### 4.1. Discretization of Equivalent Internal Work

Variation of equivalent internal work can be described as:

(16) $$\delta W_{int}=\int_{\Omega_{0}}^{}S_{IJ}\delta \epsilon_{IJ}d\Omega_{0}$$

where the variation of Green strain is given by:

(17) $$\delta \epsilon_{IJ}=0.5\left(\frac{\partial \delta u_{i}}{\partial X_{I}}F_{iJ}+\frac{\partial \delta u_{i}}{\partial X_{J}}F_{iI}\right)$$

Increments of internal work is given by linearization of Equation (16) through directional derivatives in displacement direction:

(18) $$\delta \delta W_{i}=\int^{\;}\delta \epsilon_{IJ}\delta S_{IJ}d\Omega_{0}+\int^{\;}\delta \delta \epsilon_{IJ}S_{IJ}d\Omega_{0}$$

At the first step, initial guess for increment of nodal displacements is set as zero, therefore, nonlinear terms ($K_{II}$ and $K_{III}$) would not change tangent stiffness matrix and both (linear and nonlinear) could be calculated at the same way ($K_{I}$). Difference between linear and nonlinear approaches appears at the next incremental steps of Newton–Raphson procedure. According to de Borst et al. [35], the geometric term ($K_{III}$) is important because it considers numerical instability due to geometry changes.

(19) $$\left[K_{I}+K_{II}+K_{III}\right]{\tilde{u}}^{t+\Delta t}=\delta W_{ext,i}^{t}-\delta W_{int,i}^{t}$$

### 4.2. Discretization of Equivalent External Work

Since the variation of equivalent external work is function of temperature it was necessary to calculate the temperature field through Fourier’s law. It was considered heat flow in 1-D direction with fixed boundary conditions (Equation (20)):

(20) $$\frac{\partial \theta }{\partial t}=\frac{\partial^{2}\theta }{\partial x^{2}}$$

Dimensionless functions were assumed as solution to previous equation, then, the dimensionless Fourier’s law could be written as presented in Equation (22):

(21) $$\Theta =\frac{\theta \left(x,t\right)}{\theta_{0}}\;,\;\chi =\frac{x}{{Χ}_{0}}\;\mathrm{and}\;T=\frac{\alpha \;t}{{Χ}_{0}{}^{2}}$$

(22) $$\frac{\partial \Theta }{\partial T}=\frac{\partial^{2}\Theta }{\partial \chi^{2}}$$

Using the concept of separation of variables and assuming that each function will individually satisfy the boundary condition, it is possible to rewrite a multivariable function as a sum of the product of these functions. Then, the dimensionless temperature function could be rewritten as:

(23) $$\Theta \left(T,\chi \right)=\sum^{\;}c_{i}T_{i}\left(T\right)\chi_{i}\left(\chi \right)$$

Substituting Equation (23) into Equation (22) one gets that each individual equation is related as:

(24) $$\dot{T_{i}}\chi_{i}=T_{i}\ddot{\chi_{i}}$$

Previous equality could be represented by a constant value as given in Equation (25) just rearranging Equation (24), since dimensionless time function is not related with dimensionless position function and vice-versa:

(25) $$\frac{\dot{T_{i}}}{T_{i}}=\frac{\ddot{\chi_{i}}}{\chi_{i}}=-M_{i}^{2}$$

Equations (26) and (27) can be assumed as solutions that satisfy Equation (25):

(26) $$T_{i}=C_{i}e^{-M_{i}^{2}T}$$

(27) $$\chi_{i}=A_{i}\cos\left(M_{i}\chi \right)+B_{i}\sin\left(M_{i}\chi \right)$$

Substituting Equations (26) and (27) into Equation (23) and applying the boundary restraint conditions for a nontrivial solution one can find the first unknown coefficient:

(28) $$\Theta \left(T,\chi \right)=\sum^{\;}e^{-M_{i}^{2}T}\left[{A^{\prime }}_{i}\cos\left(M_{i}\chi \right)+{B^{\prime }}_{i}\sin\left(M_{i}\chi \right)\right]$$

(29) $$\Theta \left(T=0,\chi =0\right)=0\to {A^{\prime }}_{i}=0$$

(30) $$sin\left(M_{i}\cdot 1\right)=0\to M_{i}=n\cdot \pi$$

The other coefficient can be calculated considering the boundary conditions at initial configuration:

(31) $$\Theta \left(T=0,\chi \right)=\sum^{\;}{B^{\prime }}_{i}\sin\left(M_{i}\chi \right)$$

(32) $$\int_{0}^{1}\Theta \left(T=0,\chi \right)\sin\left(M_{j}\chi \right)d\chi ={B\prime }_{i}\int_{0}^{1}\sin\left(M_{i}\chi \right)\sin\left(M_{j}\chi \right)d\chi ={B\prime }_{i}\frac{{Χ}_{0}}{2}$$

(33) $${B\prime }_{i}=\frac{2}{{Χ}_{0}}\int_{0}^{1}\Theta \left(T=0,\chi \right)\sin\left(M_{j}\chi \right)d\chi$$

Accordingly, the temperature could be calculated at each integration point just recalling Equation (21). Considering known the temperature distribution at each time t, the deformation gradient due to thermal expansion could be calculated as:

(34) $$F_{\theta }=\alpha \cdot \Delta \theta \cdot I+I$$

and the equivalent external work can be represented as function only of the thermal actions since isostatic condition is considered in this study:

(35) $$\delta W_{e}=\int^{\;}\delta \epsilon_{IJ}S{\left(\theta \right)}_{IJ}d\Omega_{0}$$

## 5. Validation and Discussion

In the first part of this section it is presented the verification of numerical formulation with analytical solutions to verify the accuracy, then comparison with results in literature are proposed. The validation tests were accomplished in two steps, one to evaluate the convergence of the nonlinear model and other to verify the numerical formulation of thermal loading. The flowchart used to code the MATLAB program is presented in Appendix A.

The nonlinear model was verified considering mechanical loads applied on a beam. Since finite displacements are assumed, the external loads used in this example are not constant, as indicated in Equation (8), because they will vary according to geometry changes. A way to solve them could be expressing the follower loads in terms of parametric variables which will be updated according to the current geometry, as indicated in Equation (36). The linearization of these surface forces must also be considered when the tangent stiffness matrix is calculated, as explained by Bonet and Wood [36].

**Figure 2:**
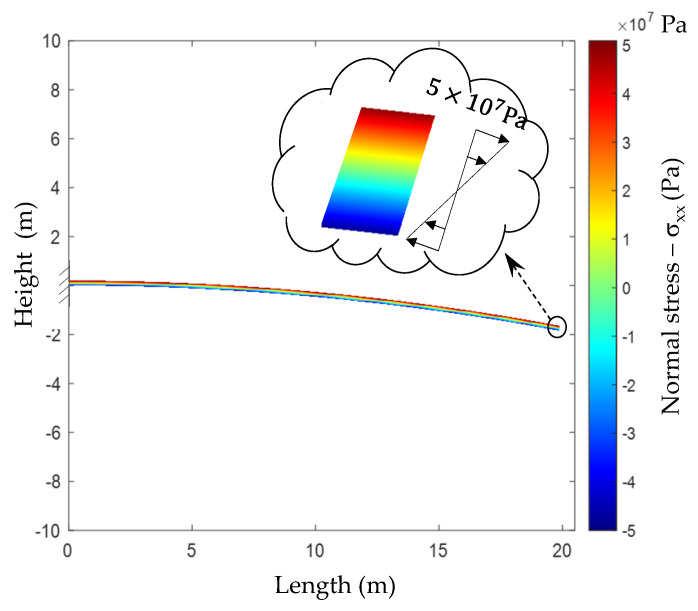
Normal stress induced by bending moment applied at the end of a built-in beam.

An example with a built-in beam with bending moment applied at the end is now considered. Since the bending moment is constant along the beam, rotated Cauchy stresses (recall Equation (4)) must remain invariant in perpendicular sections along this beam, as indicated in Figure 2.

(36) $$\delta W_{ext}=\int^{\;}N_{a}\bar{p}\left(\frac{\partial x}{\partial \xi }\times e_{3}\right)d\xi$$

Henceforth the numerical formulation of thermal stresses is considered. Thermal stresses in unrestrained slab could be calculated adding the thermal component to the Airy stress function [37,38] which, in rectangular slab in plane strain analysis, could be given by Equation (37). Since the slab is free to expand no stress is expected at the borders; hence the first term of Equation (37) corresponds to suppression of internal stresses generated due to thermal expansion (second term). The third term is related with equilibrium since asymmetric thermal distribution could result in eccentric load.

(37) $$\sigma =-\frac{E}{1-\nu }\alpha T+\frac{1}{2b}\int_{-b}^{b}\frac{E}{1-\nu }\alpha Tdx+\frac{3x}{2b^{3}}\int_{-b}^{b}\frac{E}{1-\nu }\alpha Txdx$$

**Figure 3:**
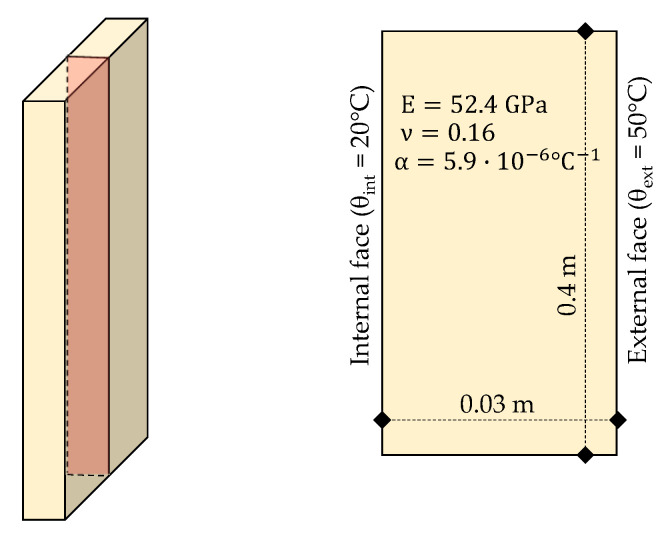
Model used in validation test.

**Figure 4:**
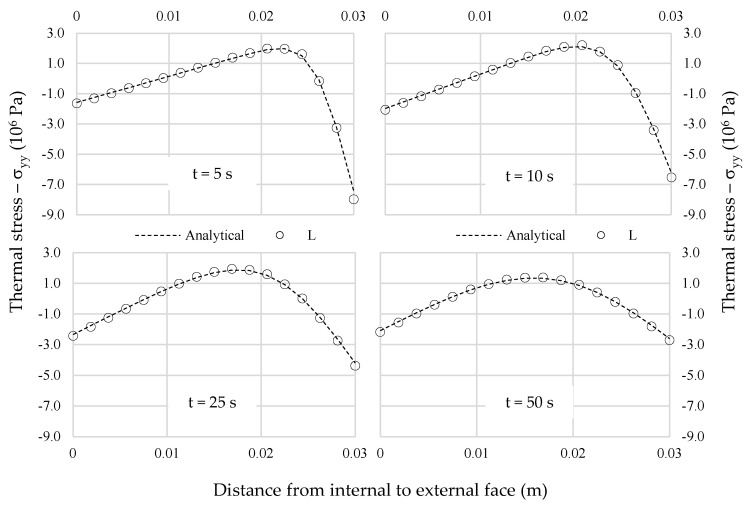
Comparison between analytical and numerical (linear) solution for several elapsed times. (t = 5 s, t = 10 s, t = 25 s, t = 50 s).

In these analyses the transient heat flux was considered with fixed boundaries conditions. The temperature on the external face was assumed as 50 °C along the whole surface, for the internal side it was assumed 20 °C. Thermal and mechanical properties of marble were assumed the same used in Ferrero et al., [9], as indicated in Figure 3. Transient heat flow was calculated to four elapsed times (5 s, 10 s, 25 s and 50 s) and good correlation was achieved between linear and analytical solution, as presented in Figure 4.

Pescara’s Building

Ferrero et al. [9] monitored the temperature of the internal and external face of a ventilated façade at the Pescara’s building during six days in 2007. An automatic system recorded the temperatures at each 15 min. The highest gradient of temperature was 4.7 °C which induced about 0.04 MPa in tensile stresses according to that model. Similar analyses were performed using the formulation presented in this work for several elapsed times to evaluate the differences between that model and the numerical approach herein proposed.

**Figure 5:**
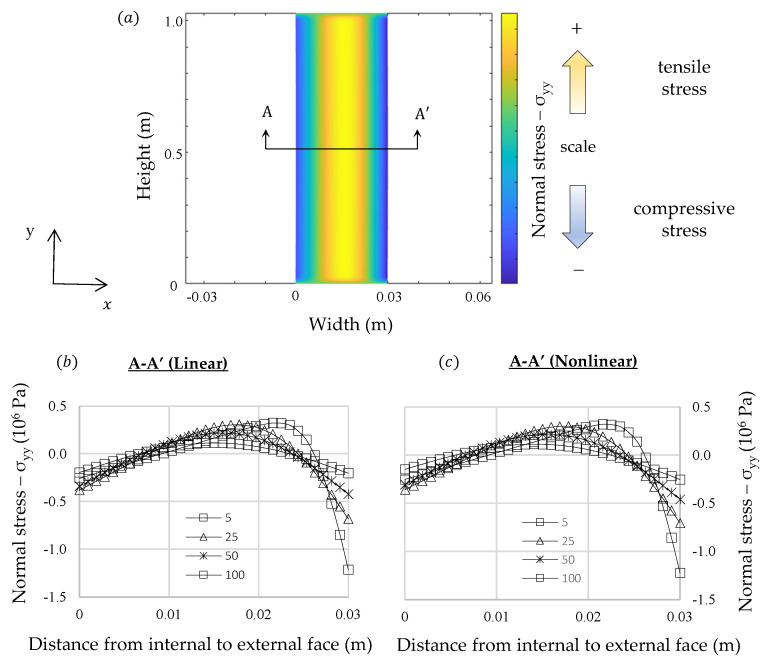
Comparison between linear and nonlinear numerical analysis of δ_yy_: (**a**) Representation of a generic normal stress distribution (**b**) linear analysis (**c**) nonlinear analysis.

**Figure 6:**
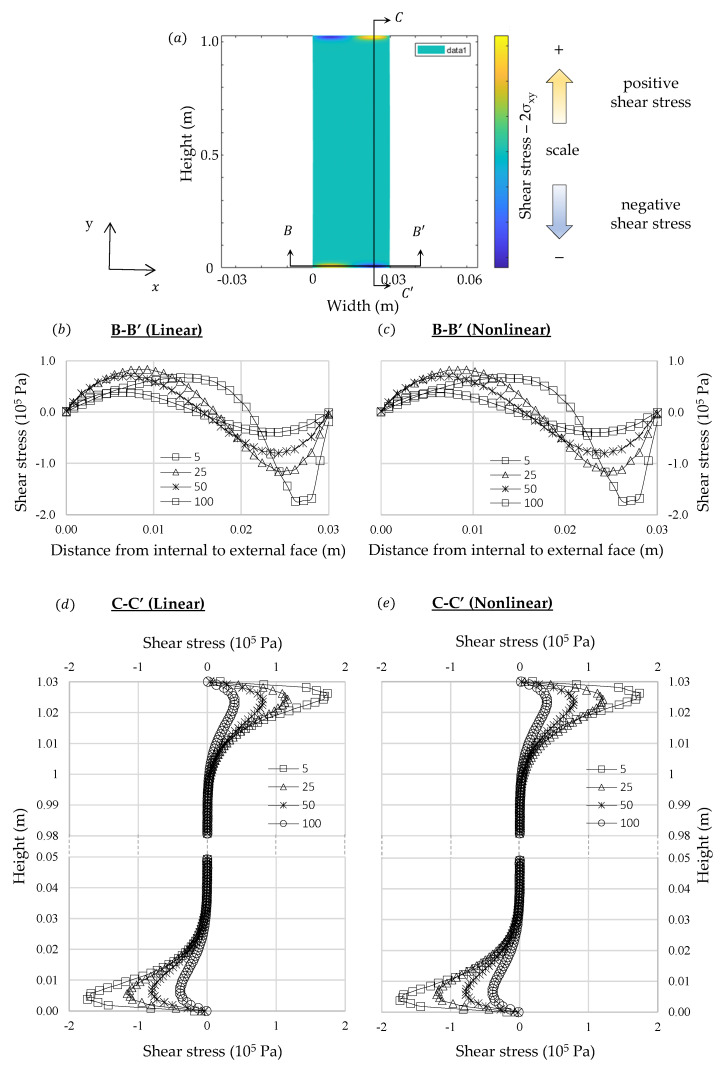
Comparison between linear and nonlinear numerical analysis of 2δ_xy_: (**a**) representation of a generic shear stress distribution, (**b**,**d**): linear analysis, (**c**,**e**): nonlinear analysis.

**Figure 7:**
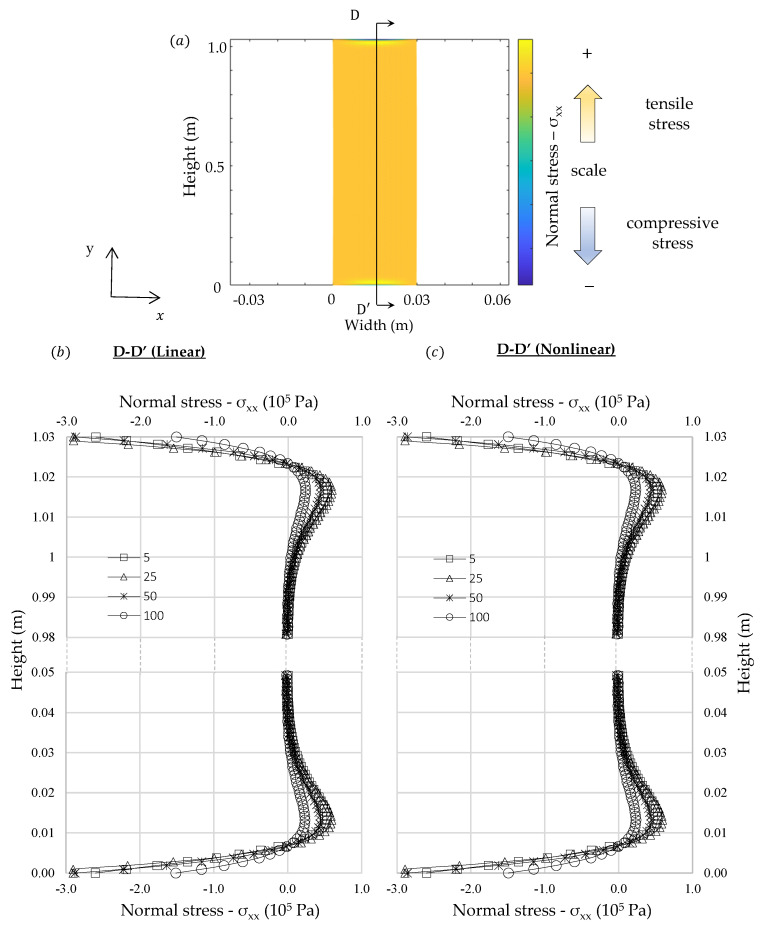
Comparison between linear and nonlinear numerical analysis of δ_xx_: (**a**) representation of a generic normal stress distribution (**b**) linear analysis (**c**) nonlinear analysis.

In Figure 5, Figure 6 and Figure 7 are presented the thermal stresses for each analysis. Normal stress distributions were considered at the symmetric planes and the shear stresses were represented in two orthogonal planes. The results show that internal stress distribution is different from the one usually found in classical beam theories, in which the traction and compression stresses belong to opposite sides of the neutral axis. Nevertheless, considering the theoretical formulation presented in the previous section it would be expected to find such a distribution because the fibers on the external face, where the thermal expansion is greater, would try to expand but they will be constrained by the adjacent fibers in which the thermal deformations are smaller. Then, the external fibers should be under compression and those on the left side would be under tensile stresses. Symmetric behavior is expected on the left-hand side.

It must be noted that internal stresses are greater at the initial instant of the transient regime and they reduce until it becomes constant in stationary condition. The results are consistent with those expected if the shape of the internal temperature curve in transient regime is considered. At the beginning only those fibers close to the right-hand side are deformed by thermal expansion, thus, greater amount of stress will be induced to counterpoise these unbalanced loads. Nonetheless, when the transient gradient approaches to stationary condition these unbalanced loads reduce. The greater stresses developed in transient analysis at the beginning could explain why the intergranular decohesion in thermal treated marble is higher close to the exposed surface, as identified by Bellopede et al. [29]. This phenomenon can also be related with the increase of bowing with the height of buildings related by Siegesmund et al. [6], since the thermal exchanges could increase according to elevation due to wind action.

Shear stresses and normal stresses in x direction were concentrated at the ends of the slab. Similar results were found by Carpinteri and Paggi [39] in nonhomogeneous beams using the classical beam’s theory, however, in that formulation shear stresses tend to infinity when they approach the boundaries. In the numerical formulation proposed in this study the shear stresses at the boundaries in simply supported slabs tend to zero.

The shear stress distribution is consistent with the theory of long thin beams, in which most of its length follow a strain profile almost translation-symmetric. At the slab edges, in which the translation-symmetry cited does not hold, there are other bidimensional deformation modes, which dissipates the energy induced by thermal strains in a more efficient way. These edge deformation modes explain why the stress calculated by the finite element method does not fit exactly to the analytical solution.

**Figure 8:**
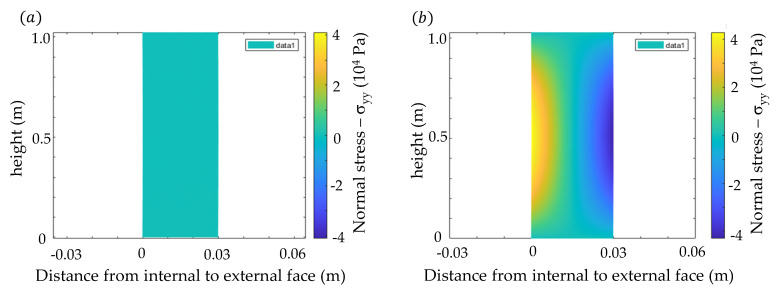
Evaluation of normal stress distribution (σ_yy_) in steady heat flux (t → ∞): (**a**) linear (**b**) nonlinear.

In transient condition the difference between stress distribution in linear and nonlinear analysis is minimal, just as expected due to the magnitude of the deformation. However, for stationary condition, the internal stresses completely vanish over the entire body in linear analysis just as expected by the analytical solution if one recalls Equation (36) considering linear distribution of temperature though width. Although, in nonlinear analysis, residual stresses still remain, as indicated in Figure 8.

## 6. Conclusions

This paper presents a numerical formulation to evaluate stresses in nonlinear thermoelastic materials. According to our model transient thermal analysis has proven to induce higher levels of stress at the beginning, i.e., at the early stages of the transient heat flux higher stresses were developed to balance the locally differential strains. These results are important to understand bowing phenomenon because transient heat flux continuously occurs in ventilated façades due to the variation of insolation and thermal exchanges between marble slabs and external environment (due to rain, wind, etc.). Therefore, even the small gradients of temperature through width can be harmful in long-term and it help to explain why the intergranular decohesion is more intense at the exposed face. The nonlinear analysis also identified remaining stresses in the slab even in stationary thermal flow. Moreover, shear stresses appear at the ends of the body, hence, for short beams or greater loads, more sophisticated models should be considered.

## Figures and Tables

**Figure A1:**
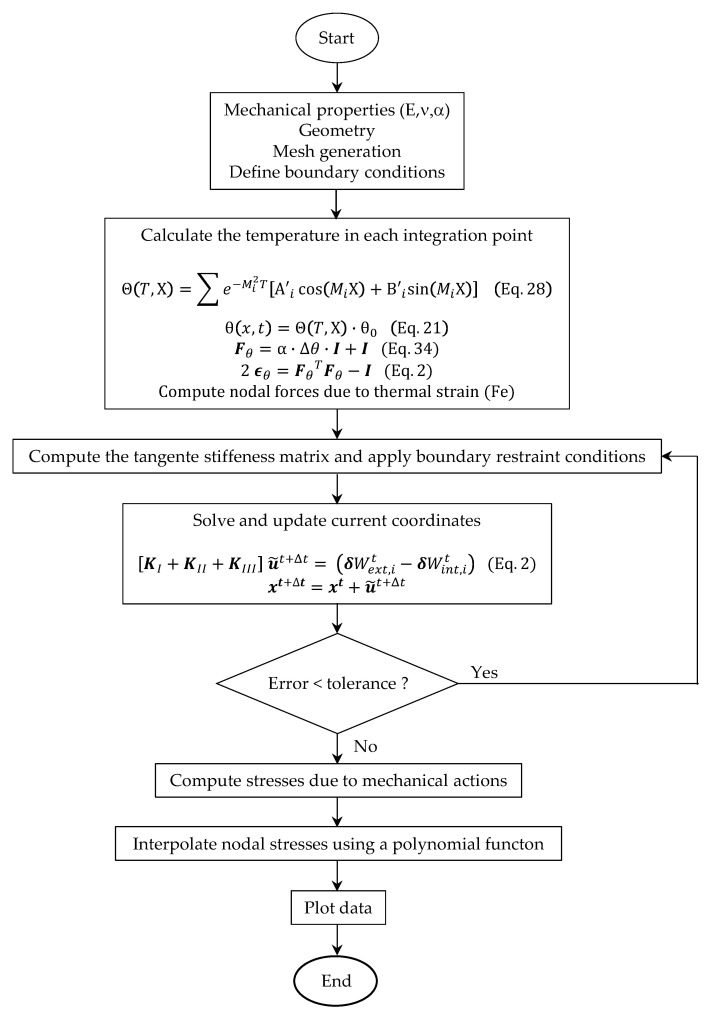
Procedure used to evaluate thermal stresses in marble slabs.

## List of Symbols

A	constant
b	slab width
B	constant
C	constitutive elastic matrix
$e_{i,x}$	orthonormal basis in the deformed configuration
$e_{i,X}$	orthonormal basis in the reference configuration
E	Young modulus
F	deformation gradient
I	identity tensor
K	stiffness matrix
M	constant
N	shape function relative to node i
$\bar{p}$	pressure load function
R	rotation tensor
S	second Piola-Kirchhoff stress tensor
t	current time
T	dimensionless time function
U	right Cauchy-Green tensor
V	left Cauchy-Green tensor
u	displacement vector
$\tilde{u}$	nodal displacement
W_ext_	external virtual work
W_int_	internal virtual work
x	coordinates in current configuration
X	Coordinates in reference configuration
α	thermal expansion coefficient
δ	Kronecker delta
δ	directional derivative
ε	engineering strain tensor
ϵ	Green strain tensor
$\epsilon_{\theta }$	strain tensor due to thermal action
$\epsilon_{mec}$	strain tensor due to mechanical action
Π	total potential energy
θ	temperature function
Θ	dimensionless temperature function
λ	eigenvalues of the tensor U
ν	Poisson ratio
σ	Cauchy stress tensor
τ	shear stress
Ω	current configuration
Ω_0_	reference configuration
${\left(\cdot \right)}_{\theta }$	relative to the thermal actions
χ	dimensionless position function

## Appendix A

**Workflow**
